# Evaluating YouTube Videos With Prophylactic Mastectomy Content in Terms of Their Quality and Reliability

**DOI:** 10.1155/tbj/9487931

**Published:** 2025-01-09

**Authors:** Tansu Altintas, Mehlika Bilgi Kirmaci

**Affiliations:** ^1^Department of General Surgery, Bahcesehir Liv Hospital, Istinye University, Istanbul, Turkey; ^2^Department of General Surgery, Afyonkarahisar Health Sciences University, Afyonkarahisar, Turkey

**Keywords:** DISCERN, GQS, prophylactic mastectomy, quality, YouTube

## Abstract

**Aim:** Purpose of this study is to investigate the quality and reliability of YouTube video contents on prophylactic mastectomy.

**Material and Methods:** The search terms “prophylactic mastectomy,” “prophylactic mastectomy surgery,” “preventive surgery for breast cancer,” “risk-reducing mastectomy,” and “prophylactic mastectomy and breast reconstruction” were searched on YouTube. The uploader, video content, length (seconds), upload date, number of days since upload date, number of views, number of comments, and likes were recorded and evaluated. Finally, the videos included in the study were evaluated using modified Quality Criteria for Consumer Health Information (DISCERN) and Global Quality Scale (GQS).

**Results:** The total number of views of the 50 videos reviewed in the study was found as 3.674.469. The mean DISCERN score of the two observers was calculated as 3.35 ± 1, and the videos were found to be of medium reliability. The mean GQS score of all videos was 3.39 ± 0.9 and the videos were of medium quality. The researchers gave 1-2 points (misleading) to 7 (14%) videos, 3 points (somewhat helpful) to 20 (40%) videos, 4 points (beneficial) to 16 (32%) videos, and 5 points (excellent) to 7 (14%) videos.

**Conclusion:** In our study, we found that the videos uploaded by doctors were of good quality, the videos uploaded by health channels were of medium quality, and the videos uploaded by patients were of poor quality and misleading. The videos with health contents should be evaluated by the relevant specialists, and only useful videos should be broadcast.

## 1. Introduction

Breast cancer is the most common cancer in women and one of the first causes of cancer-related deaths [1, 2]. Prophylactic mastectomy is a risk-reducing surgical procedure performed to reduce the risk of developing cancer in the other breast in patients with a family history of cancer, known genetic mutations such as BRCA1 or BRCA2, or who have been diagnosed with cancer in one breast. It can reduce the risk of breast cancer by up to 90-95% [3]. The most appropriate repair after protective mastectomy is simultaneous repair in the appropriate patient group. In such repairs, changes in the body image perception of the patient are reduced to minimum and a second surgical trauma is eliminated. However, since it is unlikely to completely remove the breast cells in prophylactic mastectomy and even if it does not completely prevent cancer development, surgical risks, breast structure of the patient, comorbid diseases, and esthetic expectation of the patient should be taken into detailed consideration [4]. Patients should be informed in detail about the treatment process. Internet and social media platforms allowing easy access to information today have become an unavoidable part of life [5]. According to research, 80% of the people have searched for information about their health through the Internet [6, 7].

In this point, especially YouTube (https://www.youtube.com) has become one of the most popular social platforms in the world. YouTube (YouTube LLC, San Bruno, California, United States) has more than 2.68 billion users, and more than 720,000 hours of content are uploaded to YouTube every day [8]. YouTube includes many videos on the etiology, pathogenesis, diagnosis, treatment, and prevention of numerous health problems [9–13]. YouTube is increasingly being used for health information, and it has a large information database that allows users to view, upload, and download without any price [14]. However, the use of the YouTube videos for health and medical information has a variety of disadvantages, including disorganization, complex medical language, and lack of peer review, because anyone from any part of the world can post a YouTube video that is accessible to all people without any standardization or regulation. In addition, accuracy and reliability of the information obtained may affect patients' participation in the treatment process [15]. Various studies have analyzed the effects of communicating health information via social media platforms, and especially via YouTube. However, there is no study so far to investigate the reliability of YouTube video content on prophylactic mastectomy.

Purpose of this study is to investigate the quality and reliability of YouTube video contents on prophylactic mastectomy.

## 2. Materials and Methods

### 2.1. Study Design

This observational study was designed by two general surgeon specialists (Researcher 1: female, 12 years experienced, Researcher 2: female, 13 years experienced) on 01/02/2024. Since any material taken from humans and animals was not used in the study, it was exempted from ethics committee approval [16, 17]. The search terms to be used in the study were determined using the Google Trends (https://www.trends.google.com) application [18, 19]. Accordingly, it was decided to use the search terms “Prophylactic Mastectomy,” “Prophylactic Mastectomy Surgery,” “Preventive Surgery for Breast Cancer,” “Risk-reducing Mastectomy,” and “Prophylactic Mastectomy and Breast Reconstruction” with the consensus of both Google Trends and the researchers. An Excel file was created by the researchers for the videos to be reviewed in the study. This file included the uploaders of the videos, content, length (second), view count, date of uploading, date of viewing by the researchers, the time since its upload (days), the number of daily views, the number of comments, and likes. Finally, the videos included in the study were evaluated using modified DISCERN and GQS that have been used in many studies previously [19–22].

### 2.2. Data Collection

**Figure 1:**
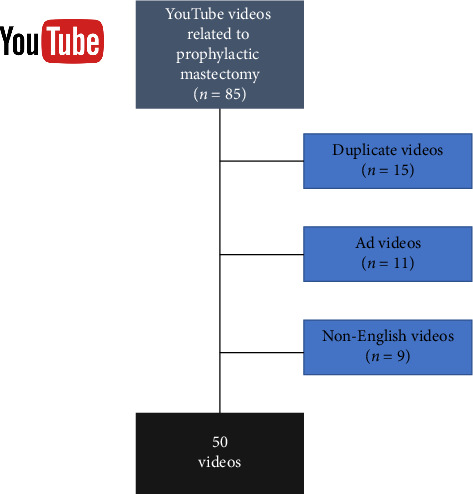
Inclusion of YouTube videos related to prophylactic mastectomy.

All search histories and cookies of the computer that will be used to determine the videos to be examined in the study were deleted and a new YouTube account was created. All search terms were filtered by selecting first “relevance” and then “view count” on YouTube. The videos that fit the sample of the study were determined by the consensus of the two researchers and saved in an Excel file. Among these videos, those with advertising content and news content, those shorter than 60 s, those with poor sound and image quality, those made for entertainment purposes, and those outside the English language were excluded from the study (Figure 1). The 50 videos suitable for the research sample were included in the study.

### 2.3. Data Evaluation

The 50 videos included in the study were determined by consensus by the researchers but were evaluated in separate settings. The structured modified DISCERN scale was used to evaluate the reliability of the examined videos and the GQS scale was used to evaluate the quality of the videos. According to the modified DISCERN scale, 1-2 points indicate poor, 3 points medium, 4 points good, and 5 points excellently reliable content [22]. Similarly, 1-2 points given to videos using the GQS scale indicate poor quality, 3 points medium quality, 4 points good quality, and 5 points excellent quality content [23].

According to modified DISCERN and GQS scale, videos are grouped by the researchers as follows: videos scored as 1-2 are “misleading,” videos scored as 3 are “somewhat helpful,” videos scored as 4 are “beneficial,” and those scored as 5 are “excellent.”

**Figure 2:**
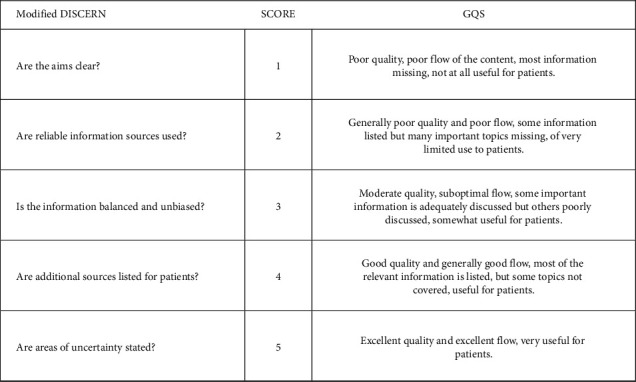
Questions of the modified DISCERN and GQS scales.

The questions of the DISCERN and GQS scales are given in Figure 2.

### 2.4. Statistical Analysis

Statistical analysis was performed using IBM SPSS Statistics Version 23.0 package software (SPSS, Statistical Package for Social Sciences, IBM Inc., Armonk, NY, USA). While evaluating the research data, frequencies (number, percentage) are given for categorical variables and descriptive statistics (mean, standard deviation) are given for numerical variables. Agreement between two independent observers was evaluated by Spearman correlation analysis and Cronbach's *α* coefficients. A value of *p* < 0.05 was considered statistically significant.

## 3. Results

The total number of views of the 50 videos reviewed in the study was found as 3.674.469. It was determined that 47 (94%) of these videos were animated videos and 3 (6%) were real images. Videos were uploaded between 2010 and 2023 and the videos were mostly uploaded in 2018 (*n* = 12). The general features of the videos are shown in Table 1.

The average daily views, video length (seconds), comments, and likes of the videos are given in Table 2.

When Table 2 is examined, it was determined that the videos uploaded by patients were watched (*p* < 0.05) more than the videos uploaded by health channels and doctors and got more comments (*p* < 0.05) and likes (*p* < 0.05) than the videos uploaded by health channels and doctors, and the difference between them was statistically significant. When it is examined according to the video contents, it was determined that patient experience videos were watched more than the other contents and more comments were made on such videos and they were liked more too. Difference between the number of daily views, comments, and likes of patient experience videos compared to the other videos is statistically significant (*p* < 0.05 for all).

The DISCERN scale was used to determine the reliability of the videos. Accordingly, the mean DISCERN score of the two observers was calculated as 3.35 ± 1, and the videos were found to be of medium reliability. The mean DISCERN score given to all videos by the first observer was 3.36 ± 1, and the mean DISCERN score of the second observer was 3.34 ± 1. According to the GQS scale used by the researchers to determine the quality of the videos they examined, the mean GQS score of all videos was 3.39 ± 0.9 and the videos were of medium quality. The mean GQS score of the first observer was 3.44 ± 0.9, while the mean GQS score of the second observer was 3.34 ± 0.9. The distribution of the scores given by the two observers according to the DISCERN and GQS scales is given in Table 3.

**Figure 3:**
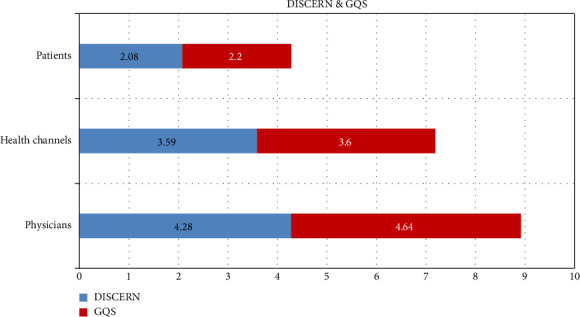
DISCERN and GQS mean scores of video uploaders.

When both DISCERN and GQS scores of the observers are evaluated, a statistically significant difference was found between the videos uploaded by doctors and the videos uploaded by both health channels (*p* < 0.05) and patient users (*p* < 0.01). Similarly, when both DISCERN and GQS scores of the observers are evaluated, a statistically significant difference was found between the videos uploaded by health channels and the videos uploaded by patients (*p* < 0.05). DISCERN and GQS mean scores given by two researchers to the videos uploaded by doctors, health channels, and patients are provided in Figure 3.

The researchers gave 1-2 points (misleading) to 7 (14%) videos, 3 points (somewhat helpful) to 20 (40%) videos, 4 points (beneficial) to 16 (32%) videos, and 5 points (excellent) to 7 (14%) videos. It has been determined that the videos uploaded by doctors and health channels are safer and higher quality than the videos uploaded by other users (Table 4).

Agreement between the two researchers has been evaluated with Cronbach's *α* coefficient. It was determined that there was an excellent agreement between the two researchers in terms of DISCERN and GQS scores (Table 5).

## 4. Discussion

YouTube is the world's most visited and largest video sharing platform. The fact that there are many health-related videos on YouTube, as well as entertaining, informative, educational videos, and that these videos can be easily accessed by everyone, has caused concern among experts [24]. Based on this, many studies evaluating YouTube videos have been conducted in the literature [11–15]. In this study, we aimed to investigate the reliability and quality of videos pertaining to prophylactic mastectomy on YouTube.

In our study, “relevant” and “most viewed” videos for prophylactic mastectomy were examined by entering five search terms separately. A total of 88 videos were examined and 50 videos suitable for the study sample were included in the study. The 50 videos we included in the study were watched 3.674.469 times in total. In a study evaluating hysterectomy videos on YouTube, 66 videos were examined and it was reported that these videos were viewed 4.679.118 times in total [25]. In a study evaluating YouTube videos and evaluating 110 videos, the total views were reported as 24.846.705 [26]. In another study where 202 YouTube videos were evaluated, the view count was reported as 2.518.512 [27]. We think that the view count of the videos is due to factors such as the incidence, diagnosis, and treatment of the diseases examined.

It was determined that 12 videos out of 50 examined (24%) were uploaded by patients and these videos were viewed 2.098.123 times. Videos uploaded by patients were viewed more and got more comments and likes than the videos uploaded by both doctors (*n* = 7) and health channels (*n* = 13). There are a lot of studies in the literature indicating that the videos uploaded by patients are more popular than the videos uploaded by health professionals [21, 28, 29]. In a study by Kuru and Erken, 50 videos were examined and it was reported that the videos uploaded by patients were more popular [12]. 50 videos with pancreas cancer content were examined by Çakmak and Mantoglu and it was reported that 7 videos uploaded by patients were viewed more than 6 million times [10].

In our study, all videos were examined by two researchers in terms of reliability with the DISCERN scale and quality with the GQS scale, and it was determined that the overall quality and safety of the videos were moderate. The two observers gave 1-2 points (misleading) to 7 (14%) videos, 3 points (somewhat helpful) to 20 (40%) videos, 4 points (beneficial) to 16 (32%) videos, and 5 points (excellent) to 7 (14%) videos. All videos containing misleading information were uploaded by non-doctor users. Similar results have been reported in previous studies. In a study by Erdogan, 50 YouTube videos were examined, and examined videos were reported to be medium quality [20]. Similarly, in their studies evaluating YouTube videos, Cakmak and Mantoglu [10] reported that the videos were of medium quality.

When we examined the videos according to the uploaders, it was determined that the videos uploaded by the doctors were generally of good quality. It was determined that the videos uploaded by health channels were of medium quality. However, even in the videos uploaded by health channels, it was seen that incomplete information was provided or clinical advertisements were made. There are studies in the literature reporting that videos uploaded by health channels are partially beneficial [13, 14, 20].

Previous studies have reported that videos with health content should only be uploaded by healthcare specialists [13, 26]. We also agree with this opinion and we think that these videos should be updated periodically after they are uploaded by medical specialists and that the videos should be evaluated by expert referees before they are published. In our study, it was determined that the videos uploaded by health channels were of medium quality. It has been observed that these videos generally contain clinical advertisements and incomplete and misleading information. The videos uploaded by other users were found to contain poor quality and misleading information. In similar studies, it has been reported that the videos uploaded by patients and their relatives are misleading and these videos are viewed more than useful videos [12, 13]. In a study in which the videos with the content of postsurgical breast cancer were examined, 82 videos were examined, and it was reported that 51.2% of these videos were misleading [30]. Again, in a study in which 50 videos with breast cancer content were examined, it was reported that 86% of the videos contained misleading information [13]. In our study, only 12 videos were uploaded by the patients, and 8 of these videos were considered to be of poor quality and 4 of them were considered to be of medium quality by both observers.

In our study and many other studies, only English videos were evaluated. In a study conducted by García-Cano-Fernández et al., 50 Spanish videos on bladder cancer on YouTube were analyzed and it was reported that 30.8% of the videos were of moderate/good quality and 66.7% of the videos were of poor quality [31]. In a study conducted in India, 50 Hindi videos on YouTube with breast cancer content were evaluated and it was reported that the videos were of moderate quality [32]. We think that non-English health videos on YouTube should also be examined.

### 4.1. Study Limitations

First, it should be kept in mind that data of this study were obtained at a specific time because the parameters of YouTube videos change every minute. Second, only English videos were included. The fact that the number of the videos on prophylactic mastectomy is limited and most of these videos are included in the study forms the powerful side of the study.

## 5. Conclusion

In our study, we found that the videos uploaded by doctors were of good quality, the videos uploaded by health channels were of medium quality, and the videos uploaded by patients were of poor quality and misleading. It is difficult to control the videos uploaded by patients, but here we can give advice to healthcare professionals. We believe that health professionals should increase the number of comprehensive, understandable, and informative videos explaining the diagnosis, treatment, and possible complications of the disease. In addition, more videos created by doctors together with patients who have experienced the disease process can help people access accurate and useful information. Finally, the videos with health contents should be evaluated by the relevant specialists and only useful videos should be broadcast.

## Figures and Tables

**Table 1:** General features of the videos.

	*n* (%)	View	Comment	Like
*Image*				
Real	47 (94%)	3.499.377	3.913	28.552
Animation	3 (6%)	178.000	59	1.538

*Uploaders*				
Physician	7 (14%)	442.190	167	1.995
Health channel	31 (62%)	1.134.065	434	6.023
Patients	12 (24%)	2.098.123	3.371	22.072

*Content*				
General info	20 (40%)	840.534	484	6.034
Surgical technique	4 (8%)	259.500	38	612
Postoperative care	5 (10%)	277.674	18	583
Patient experience	21 (42%)	2.296.269	3.432	22.861

**Table 2:** Average distribution rates of videos according to their general characteristics.

Feature	Daily views	Video length	Comments	Likes
	Mean ± SD			
*Image*				
Real (*n* = 47)	37.39 ± 127.87	388.70 ± 276.73	85.01 ± 194.37	607.48 ± 2646.7
Animation (*n* = 3)	31.7 ± 3.1	546.7 ± 185.7	19.7 ± 14.3	512.7 ± 26.1

*Uploaders*				
Health channels (*n* = 31)	14.8 ± 127.9	355.7 ± 247.7	14 ± 390	194.3 ± 2646.7
Patients (*n* = 12)	97.5 ± 259.1	546.7 ± 292.2	280.9 ± 794.7	1839.4 ± 5408.6
Physician (*n* = 7)	31.8 ± 36.7	331.4 ± 269.5	23.9 ± 38.4	285 ± 357.8

*Video content*				
Patient experience (*n* = 21)	60.1 ± 197.3	450.5 ± 282.5	163.5 ± 605.6	1088.6 ± 4109.7
General info (*n* = 20)	22.3 ± 30.8	412 ± 301.2	24.2 ± 47.3	301.77 ± 436.1
Postoperative care (*n* = 5)	15.6 ± 7.8	177.8 ± 55.4	3.6 ± 5.5	116.7 ± 41.3
Surgical technique (*n* = 4)	16.8 ± 21.5	330 ± 199	9.5 ± 10	153 ± 161.6

**Table 3:** DISCERN and GQS scores given by the observers.

	*n* (%)	DISCERN		GQS	
		1. Researcher	2. Researcher	1. Researcher	2. Researcher
*Image*					
Real	47 (94%)	3.3 ± 1	3.2 ± 1	3.4 ± 1	3.3 ± 1
Animation	3 (6%)	4.4 ± 1	4 ± 0	4.3 ± 0.6	4 ± 1

*Uploaders*					
Health channels	31 (62%)	3.6 ± 0.7	3.5 ± 0.6	3.7 ± 0.5	3.5 ± 0.6
Patients	12 (24%)	2.1 ± 0.8	2.1 ± 0.9	2.2 ± 0.8	2.3 ± 0.7
Physician	7 (14%)	4.4 ± 0.5	4.2 ± 0.7	4.6 ± 0.5	4.7 ± 0.4

*Video content*					
Patient experience	21 (42%)	2.7 ± 0.9	2.6 ± 0.9	2.8 ± 1	2.8 ± 0.9
General info	20 (40%)	3.9 ± 0.8	3.7 ± 0.7	3.85 ± 0.7	3.8 ± 0.8
Postoperative care	5 (10%)	3.8 ± 0.8	4 ± 0.7	4 ± 0.7	3.8 ± 0.1
Surgical technique	4 (8%)	3.7 ± 0.9	4 ± 1	4 ± 0.8	3.8 ± 1

**Table 4:** Classification of the scores given by the researchers to the videos.

	Misleading		Somewhat helpful	Beneficial	Excellent
	1 score	2 score	3 score	4 score	5 score
*DISCERN*					
1. Researcher	3	4	20	16	7
2. Researcher	4	3	21	16	6

*GQS*					
1. Researcher	3	4	20	17	6
2. Researcher	4	3	20	16	7

**Table 5:** Agreement between two researchers.

	Mean ± SD	*p*	*r*	Cronbach's *α*
*DISCERN*				
1. Researcher	3.36 ± 1.04	**p** < 0.01	**0.949**	**0.933**
2. Researcher	3.34 ± 1.01			

*GQS*				
1. Researcher	3.44 ± 0.99	**p** < 0.01	**0.954**	**0.899**
2. Researcher	3.34 ± 0.98			

*Note:* Bold values represent statistically significant and in agreement between the two researchers.

## Data Availability

The data that support the findings of this study are available on request from the corresponding author. The data are not publicly available due to privacy or ethical restrictions.
